# Occurrence of Selected Pharmaceuticals in the East London Coastline Encompassing Major Rivers, Estuaries, and Seawater in the Eastern Cape Province of South Africa

**DOI:** 10.1021/acsmeasuresciau.4c00004

**Published:** 2024-04-12

**Authors:** Ronewa Netshithothole, Lawrence Mzukisi Madikizela

**Affiliations:** Institute of Nanotechnology and Water Sustainability, University of South Africa, Private Bag X6, Florida 1710, South Africa

**Keywords:** pharmaceuticals, marine and coastal environment, health risk assessment, contamination, suspect screening

## Abstract

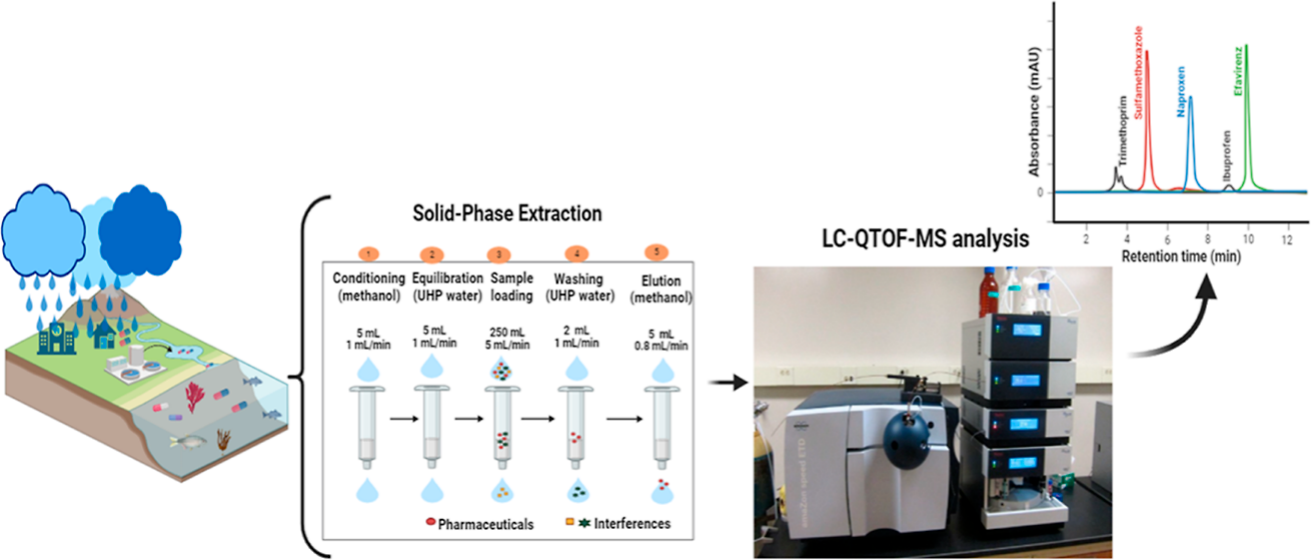

This study investigated the occurrence of ibuprofen, naproxen, sulfamethoxazole, trimethoprim, and efavirenz in water resources (river, estuarine, and sea waters) of the East London coastline, South Africa. These pharmaceuticals were previously reported to be dominant in wastewater and inland rivers of South Africa. Hence, it is important to monitor their occurrence in the coastal and marine environment. The pharmaceuticals of interest were extracted with a solid-phase extraction method and analyzed by using a liquid chromatography-quadrupole time-of-flight mass spectrometry instrument. The analytical method was validated by spiking the environmental samples with a mixture of pharmaceuticals at two concentration levels (5 and 15 μg L^–1^). The analytical method yielded acceptable recoveries ranging from 75 to 107%, with method quantitation limits from 0.16 to 9.44 ng of L^–1^. All five targeted pharmaceuticals were detected in seawater samples, with ibuprofen recording the highest concentration of 90 ng L^–1^. However, it was efavirenz and sulfamethoxazole with the highest concentrations of 572 and 60 ng L^–1^, respectively, in the Gonubie River that showed high ecotoxicological risks toward the aquatic organisms. There were no risks associated with the occurrence of other targeted pharmaceuticals. The suspect screening showed the occurrence of 57 additional pharmaceuticals in samples, with antibiotics being more dominant. The results of the present study demonstrate a need to perform a more robust investigation on the occurrence of a wide range of pharmaceuticals along the South African coasts.

## 1 Introduction

The constant presence of pharmaceuticals in water systems around the world has induced great interest in environmental scientists and analytical chemists due to a need to establish suitable analytical methods for their analysis and to perform continuous monitoring of these drugs in the environment. Pharmaceuticals of different therapeutic groups have been found present in large quantities in wastewater with wastewater treatment plants (WWTPs) being flagged as the main source of these chemicals in the aquatic environment.^1^ Hence, various pharmaceuticals are continuously released with wastewater effluents to nearby rivers and make their way to the coastal environment. Owing to the limited scientific information on the monitoring of pharmaceuticals in the environment, the environmental fate of these chemicals is poorly understood. Thus, so far, the available information shows the spread of pharmaceuticals in water resources,^2^ aquatic plants,^3^ and organisms.^4^ Globally, pharmaceuticals have been more monitored in water resources, thus resulting in overlooking other environmental matrices. Although surface water contamination with pharmaceuticals seems to be a hot research topic, there are still limited studies performed on the analysis of these chemicals in seawater.

The presence of pharmaceuticals in waterbodies found outside the African borders was already reported in the early 1980s.^5^ But it took at least two additional decades before the same chemicals were measured in the African water systems. The research on environmental monitoring of pharmaceuticals in South Africa has become more intense over the past decade.^6^ In this case, pharmaceuticals that gained more interest were those belonging to the therapeutic classes of antibiotics, antiretroviral drugs, and nonsteroidal anti-inflammatory drugs (NSAIDs). In South Africa, these pharmaceuticals are mostly monitored in wastewater and surface water. Some studies have investigated the occurrence of pharmaceuticals in river sediments and sewage sludge. It has been established that pharmaceuticals exist in surface water, and they are being carried through river water into the estuaries. In this case, mixtures of pharmaceuticals have been detected near the mouth of some rivers.^2,7^ This showcases the possibility of releasing pharmaceuticals into the seawater. Marine outfalls have also been linked as potential sources of pharmaceuticals in the marine environment.^8^ Since the ocean is a big waterbody, marine outfalls can only contribute to the occurrence of pharmaceuticals in seawater in geographical locations that contain such infrastructure.

Research focusing on investigating the occurrence of pharmaceuticals in marine and coastal environments has intensified in recent years.^9^ Indeed, concoctions of pharmaceuticals and their metabolites have been detected in seawater.^9^ This means that humans are unintentionally exposed to pharmaceuticals, with a likelihood of consuming these drugs unplanned. Reports on the occurrence of pharmaceuticals in marine organisms and seafood have also emerged in recent years,^10^ indicating the potential risks that could arise due to the continuous consumption of such food sources. Despite the availability of such information, studies on the monitoring of pharmaceuticals in seawater remain scanty. The lack of environmental monitoring studies, especially those focusing on the determination of pharmaceuticals in seawater, is more severe in Africa. The African continent has always been lagging in addressing the issues related to the environmental monitoring of emerging contaminants.

In comparison with other African countries, South Africa has conducted more research focusing on the occurrence of pharmaceuticals in waterbodies, more particularly wastewater and surface water.^11^ This study focused on investigating the occurrence of efavirenz (antiretroviral), naproxen, ibuprofen (both NSAIDs), trimethoprim, and sulfamethoxazole (both antibiotics). These pharmaceuticals are the most prominent drugs in the South African waters, with their monitoring and detection being done mostly in wastewater and river water. This creates knowledge gaps in relation to limited information on the occurrence of these pharmaceuticals in the coastal and marine environment. Thus, resulting in an inability to establish the fate of these pharmaceuticals in the environment, the coastal and marine environments are mostly ignored during the environmental monitoring studies. Therefore, the aim of this study was to investigate the occurrence of these pharmaceuticals in rivers, estuaries, and seawaters, followed by evaluating their ecological risks to aquatic organisms. This was necessary as these pharmaceuticals have been constantly detected in the South African wastewater and river water. In addition, suspect screening was performed to tentatively establish other pharmaceuticals that could be present in the investigated water resources.

## 2 Experimental Section

### 2.1 Chemicals and Materials

Pharmaceuticals: efavirenz (99.8%), ibuprofen (99.6%), naproxen (≥98%), sulfamethoxazole (≥98%), and trimethoprim (99.8%) were procured in the powder form from Merck Chemicals (Pty) Ltd. (Johannesburg, South Africa). Solvents used in this study were acetonitrile (99.9%), formic acid (98%), and methanol (99.9%), all of which were of LC–MS grade and purchased from Merck Chemicals. Ultrahigh-purity water was generated in our laboratory located in the Florida Science Campus of the University of South Africa. The Oasis HLB 6 cm^3^/150 mg solid-phase extraction (SPE) cartridges from Microsep (Johannesburg, South Africa) were used to extract targeted pharmaceutical residues from the investigated water samples.

### 2.2 Sampling and Sample Pretreatment

**Figure 1:**
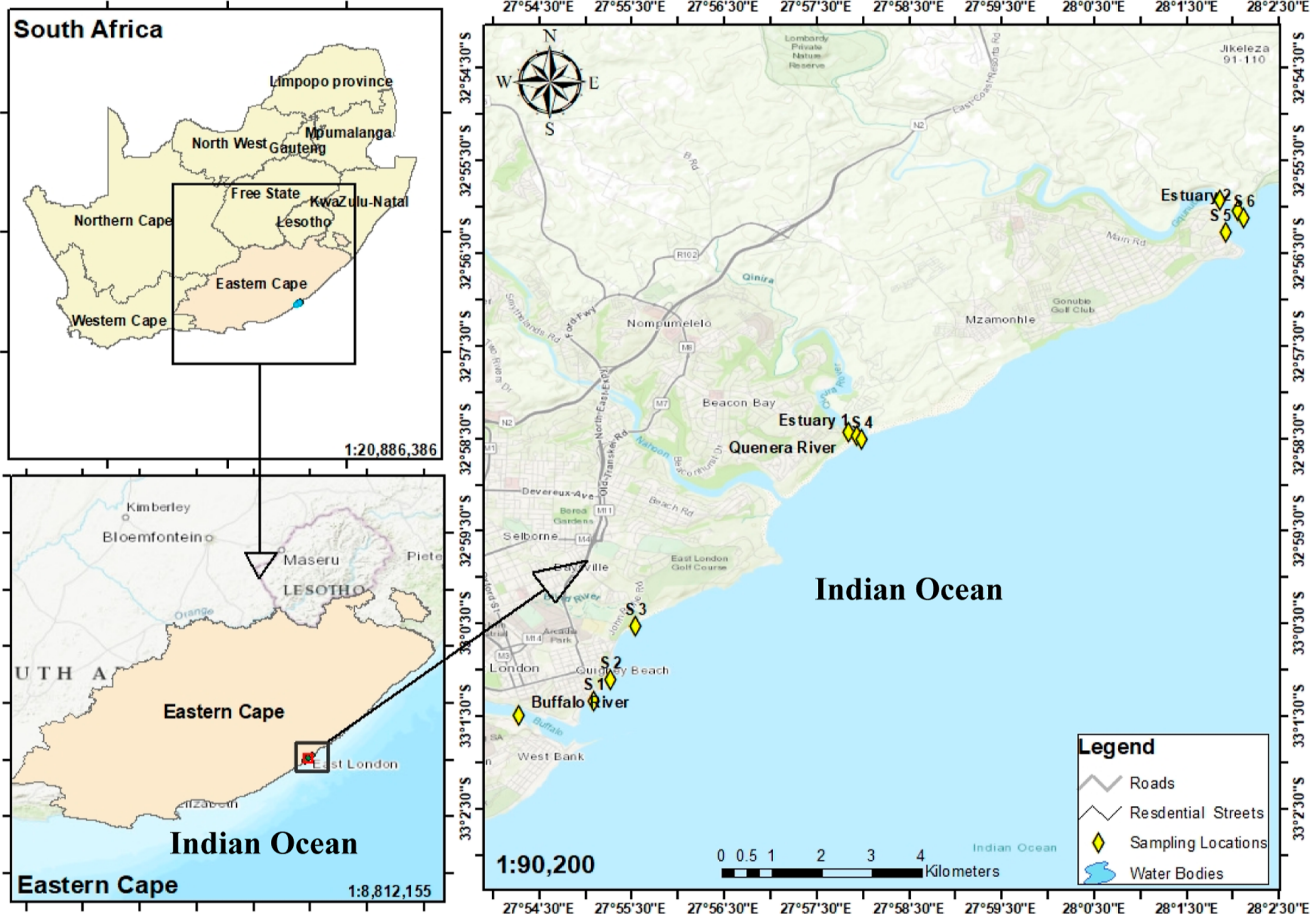
Sampling sites along the coastline of East London, South Africa.

Water samples were collected from the major rivers and their estuaries found around the city of East London in the Eastern Cape Province of South Africa using precleaned glass bottles. Seawater samples were collected along the coast of the same city, incorporating water from the swimming areas of this coastline. Figure 1 shows the sampling sites in this study area. The sampling site in the Buffalo River is near its estuary (estuary not accessible for sample collection) within the city. The sampling of the Buffalo River in this location was of interest considering the potential inputs of various domestic and industrial chemicals via different sources. The river flows through King William’s Town and near the major residential areas such as Zwelitsha and Mdantsane before it reaches East London where it pours into the Indian Ocean. Notably, various domestic WWTPs are in the proximity of the river and discharge their effluents into the river. Consequently, pharmaceuticals and other chemicals have been detected in the Buffalo River.^12,13^ As a result, the measurement of chemicals in the East London coastline becomes an important task to execute. Other rivers under study were the Gonubie and Quenera rivers, which have no information related to their contamination in the literature (to the best of our knowledge). The estuaries of these two rivers serve as recreational sites with camping activities on their banks. Seawater samples were collected along the coastline between the estuaries of Buffalo and Gonubie rivers.

The collected samples were transported to the laboratory using cooler boxes packed with ice cubes. These samples were filtered through the 125 mm Whatman grade 1 filter papers from Merck Chemicals (Pty) Ltd. (Johannesburg, South Africa) and subjected to extraction using the SPE approach.

### 2.3 Solid-Phase Extraction

The extraction and preconcentration of the investigated pharmaceuticals in water samples were executed using the automated Dionex AutoTrace 280 SPE system sourced from Thermo Fisher Scientific (Waltham, United States). About 250 mL of each filtered water sample was percolated onto the preconditioned Oasis HLB 6 cm^3^/150 mg SPE cartridge at a flow rate of 5 mL min^–1^. The conditioning of the SPE cartridge was performed with 5 mL of methanol and equilibrated with 5 mL of water flowing at 1 mL min^–1^. After the percolation of the water sample, the cartridge was rinsed with 2 mL of ultrahigh purity water flowing at 1 mL min^–1^. This was followed by drying the cartridge for 5 min with a moderate stream of nitrogen gas. Thereafter, 10 mL of methanol was used to elute the retained analytes at a flow rate of 0.8 mL min^–1^. Methanol was removed from the sample vial through vaporization and reconstituted in 1 mL of 0.1% formic acid in methanol. This solution was then subjected to a liquid chromatographic system equipped with a mass spectrometry detector (LC–MS) for analysis.

### 2.4 Chromatographic Analysis

The identification and quantification of the selected pharmaceuticals in the extracts of water samples were achieved using the Dionex Ultimate 3000 ultrahigh performance liquid chromatography (UHPLC) system from Thermo Fisher Scientific (Waltham, MA, USA). The chromatographic instrument was coupled to an Impact II quadrupole time-of-flight (QTOF) tandem mass spectrometer detection system equipped with electrospray ionization from Bruker (Bremen, Germany). The chromatographic separation of investigated compounds was performed using an XBridge C8 (3.5 μm × 3 mm × 100 mm) analytical column purchased from Waters Corporation (Milford, MA, United States). The chromatographic separation was performed while the column was housed at 30 °C. The optimum separation of investigated pharmaceuticals was achieved utilizing the mobile phase which consisted of 0.1% formic acid in ultrahigh purity water (solvent A) and 0.1% formic acid in acetonitrile (solvent B) operated in multistep gradient elution mode. The gradient elution began with 2% of solvent B for 1.5 min, increased to 10% for 2.5 min, to 50% for 6 min, and to 100% for 2 min, and then returned to the initial conditions. The sample injection volume and mobile phase flow rate were set to be 5 μL and 0.300 mL min^–1^, respectively. The positive electrospray mode was used to detect all analytes, and the Bruker QUANT analysis software was used for data processing.

### 2.5 Method Validation

The applied analytical method was validated for its ability to sufficiently extract the target compounds from water samples, followed by analysis using the employed chromatographic technique. This was done by spiking the collected water samples with a mixture of investigated pharmaceuticals at two spiking concentrations, 5 and 15 μg L^–1^. The spiked water solutions were subjected to SPE followed by performing the analysis with a described chromatographic system. Unless stated otherwise, all experiments were performed in triplicate. The validation of the analytical method was based on computing several parameters, which included the method detection (MDL) and quantitation limits (MQL) that were used as the measure of the sensitivity of the analytical method. MDL and MQL were measured as the concentrations where the signal-to-noise ratios were 3 and 10, respectively. Other investigated parameters were analyte recoveries, relative standard deviations (RSDs), and the influence of the matrix effects. In this case, the analyte recoveries and RSD values were used to quantify the accuracy and precision of the analytical method, respectively. Computation of matrix effects was based on eq 1 which considered the slopes of matrix-matched calibration (*S*_matrix_) and the solvent standard calibration (*S*_solvent_) as described elsewhere^14^
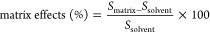
1

### 2.6 Screening of Pharmaceuticals in Samples

In addition to the five targeted drugs, the tentative identification of other pharmaceuticals present in environmental samples was performed. This was done by following a method described by Ncube and his co-workers with slight modifications.^15^ Briefly, the mass spectrometry data files were first converted to mzXML by using MSConvert software. The mzXML files were then assessed using MZmine 2.53 software.^16^ The processing settings of MZmine 2.53 were configured in compliance with a previous report, which used centroid techniques that included mass detection, chromatograph building, and peak deconvolution.^15^ The aligned peak list, which contained the *m*/*z*, retention time, and peak heights in each sample, was exported in CSV format. The exported file was then loaded into online databases (KEGG and Mass Bank) for compound identification. In all cases, the only considered match with the compounds in the database was greater than 80%. Thereafter, the identified compound was then extracted from the sample chromatogram data for further confirmation.

## 3 Results and Discussion

### 3.1 Quality Assurance

**Figure 2:**
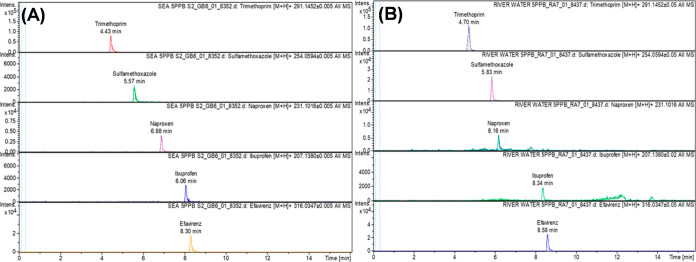
Chromatograms of the selected pharmaceuticals after spiking seawater (A) and river water (B) samples (spiking concentration = 5 μg L^–1^).

**Figure 3:**
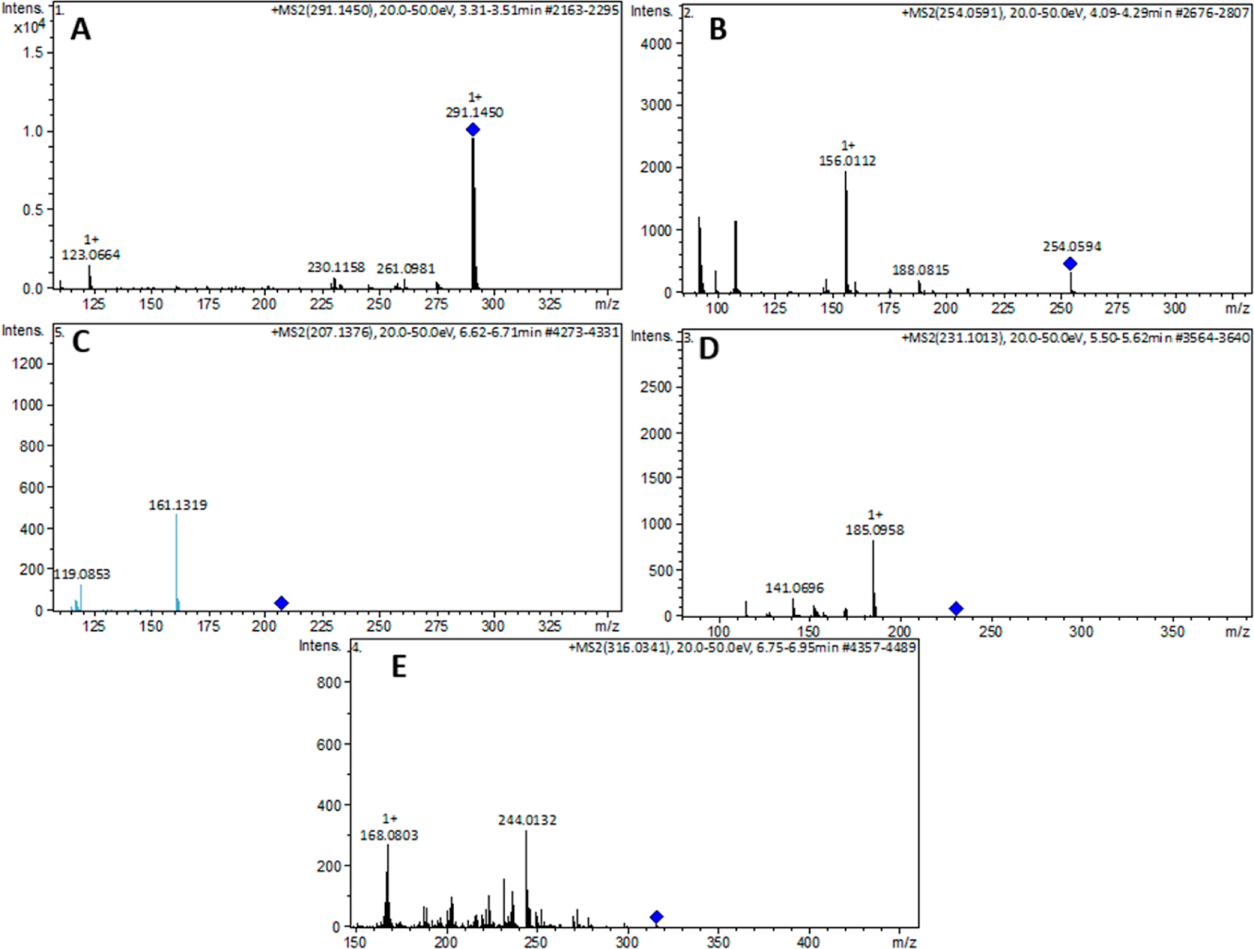
MS/MS spectra and plausible fragmentation of trimethoprim (A), sulfamethoxazole (B), ibuprofen (C), naproxen (D), and efavirenz (E).

The quality of the analytical method was validated by spiking the deionized water and real samples (river water and seawater) to establish the influence of the matrix on the analytical method. Chromatograms resulting from the spiked real samples are presented in Figure 2, with fragmentation patterns provided in Figure 3. These results show that despite the expected complexity that could be presented by the river water and seawater samples on chromatographic analysis, the peaks corresponding to the analytes are well defined. This could probably be a result of a suitable sample preparation procedure which was capable of eliminating some of the matrix effects. Results in Table 1 further show that the matrix effects were generally tolerable, showing signal enhancement for all compounds ranging from 2 to 28%, except for naproxen, which gave 60% in river water. Taking these results into account where the matrix was found to result in signal enhancement, matrix-matched calibrations were performed for all compounds as opposed to applying the external calibration curves for quantitative analysis. This approach yielded accurate results with recoveries of 75–107% attained over two spiking concentration levels of 5 and 15 μg L^–1^ (Table 1). A previous study showed that the addition of salt (up to 4% (m/v)) into aqueous samples prior to SPE performed with Oasis HLB cartridges still results in recoveries exceeding 80% for both naproxen and diclofenac.^17^ This means that the quantity of the salt content expected in the coastal environment has a minimum contribution to the accuracy of the applied analytical method toward these analytes. Indeed, acceptable recoveries were found for both river water and seawater sampled in the present study. The RSD values were below 15% indicating the precision of the applied analytical method. The method detection and quantitation limits are presented in Table 1. The presented analytical method was then applied for the quantitative determination of the five pharmaceuticals in river, estuarine, and seawater samples.

**Table 1:** Correlation Coefficients, Method Detection Limit, Method Quantification Limit, Average Recoveries (%, *n* = 3), and Relative Standard Deviation of the Selected Pharmaceuticals in Samples Collected from East London, South Africa^a^

pharmaceutical	*R*^2^	deionized water		river water					seawater				
		recovery ± % RSD		recovery ± % RSD		MDL (ng L^–1^)	MQL (ng L^–1^)	matrix effect (%)	recovery ± % RSD		MDL (ng L^–1^)	MQL (ng L^–1^)	matrix effect (%)
		5 μg L^–1^	15 μg L^–1^	5 μg L^–1^	15 μg L^–1^				5 μg L^–1^	15 μg L^–1^			
ibuprofen	0.9886	75 ± 8	94 ± 13	77 ± 7	75 ± 4	3.12	9.44	18	86 ± 5	89 ± 1	2.16	6.56	19
naproxen	0.9483	99 ± 3	103 ± 4	92 ± 3	95 ± 6	2.72	8.20	60	82 ± 12	75 ± 4	1.04	3.12	28
sulfamethoxazole	0.9981	103 ± 1	102 ± 1	91 ± 5	92 ± 4	0.16	0.48	9	89 ± 8	85 ± 2	1.64	4.93	5
trimethoprim	0.9949	107 ± 3	98 ± 2	75 ± 3	83 ± 5	0.04	0.16	28	89 ± 7	86 ± 4	0.04	0.20	6
efavirenz	0.9907	96 ± 4	89 ± 1	79 ± 6	87 ± 5	0.12	0.32	4	82 ± 4	75 ± 2	0.84	0.72	7

a The standard concentration range used in each calibration was 10–1000 μg L^–1^.

### 3.2 Occurrence of Selected Pharmaceuticals in Seawater

The previous detections of pharmaceuticals in South African oceans and seafood such as those in Durban^17^ and Cape Town^18,19^ demand a countrywide screening of these chemicals in the coastal and marine environment. In recent years, several pharmaceuticals were detected in water and sediments from the Buffalo River near the Indian Ocean,^13,20^ which prompted an investigation into the occurrence of these chemicals in the estuary of this river and the nearby seawater. Results of the present investigation are listed in Table 2. As shown in Table 2, all five investigated pharmaceuticals were detected in river water samples, with trimethoprim and ibuprofen being detected in all three rivers. Despite the constant detection of these two analytes in river water, efavirenz was the pharmaceutical that had the highest concentration of 572 ng L^–1^ in the Gonubie River. It was further found to be present in both estuaries. Ibuprofen was detected at concentrations below its method quantitation limit in river water samples, except in the Quenera River where it was found to reach 461 ng L^–1^. In the estuaries, naproxen was not detected. Ibuprofen had the highest concentration of 71 ng L^–1^ recorded in the Quenera estuary. In fact, ibuprofen proved to be more prevalent in the estuarine water as its concentration was 55 ng L^–1^ in the Gonubie estuary. Sulfamethoxazole had high levels in estuaries as well, as it was detected with a combined concentration of 75 ng L^–1^. The prevalence and detection of higher levels of ibuprofen and sulfamethoxazole are common in South African waters.^11,15^

**Table 2:** Concentrations of the Investigated Pharmaceuticals in Water Samples^a^

sample	average detected concentration (ng L^–1^) ± standard deviation (*n* = 3)				
	NSAIDs		antibiotics		antiretroviral
	ibuprofen	naproxen	sulfamethoxazole	trimethoprim	efavirenz
River					
Buffalo River	<LOQ	<LOQ	Nd	0.934 ± 0.100	Nd
Quenera River	461 ± 0.068	25 ± 0.001	23 ± 1.002	<LOQ	Nd
Gonubie River	<LOQ	Nd	60 ± 0.001	2 ± 0.524	572 ± 0.034
Estuaries (mixing point)					
Quenera River estuary	71 ± 5.50	Nd	57 ± 0.013	4 ± 0.290	48 ± 0.024
Gonubie River estuary	55 ± 0.687	Nd	17 ± 0.000	3 ± 0.311	9 ± 0.003
Seawater					
site 1	90 ± 2.549	11 ± 0.979	22 ± 0.006	2 ± 0.248	16 ± 0.012
site 2	52 ± 0.888	7 ± 0.693	6 ± 0.003	15 ± 0.771	76 ± 0.002
site 3	55 ± 1.812	Nd	25 ± 0.003	1 ± 0.064	17 ± 0.002
site 4	56 ± 2.979	10 ± 1.189	13 ± 0.002	2 ± 0.186	15 ± 0.001
site 5	66 ± 1.083	19 ± 0.524	30 ± 0.01	6 ± 0.342	25 ± 0.012
site 6	64 ± 1.117	57 ± 0.220	11 ± 0.003	4 ± 0.191	46 ± 0.022

a Nd; not detected.

In all seawater samples, the investigated pharmaceuticals were detected except naproxen, which was not found in site 3. Distribution of investigated pharmaceuticals in the beaches around East London was observed. This observation could be influenced by the fact that some study sites (sites 1–3) are within the East London Central Business District, encompassing popular beaches where locals and visitors spend their free time. This increases the chances of the direct disposal of medication into the sea and excretion of human waste along the coast with the possibility of pharmaceuticals seeping into the seawater. Furthermore, study site 1 is near the estuary of the Buffalo River which is already known for its contamination with pharmaceuticals.^13,20^ A different South African-based research has already observed that the pharmaceutical occurrence in seawater is more pronounced near the contaminated estuaries.^17^ Among the investigated pharmaceuticals, ibuprofen, sulfamethoxazole, and efavirenz are the drugs that had mostly higher concentrations in seawater when compared to the levels found for naproxen and trimethoprim. Although this is the case, naproxen concentration reached 57 ng L^–1^ in site 6. But the other NSAID, ibuprofen, prominently appeared with higher concentrations in seawater ranging from 52 ng L^–1^ (site 2) to 90 ng L^–1^ (site 1). The maximum concentration found for efavirenz was 76 ng of L^–1^ (site 2). For antibiotics, sulfamethoxazole had higher concentrations (6–30 ng L^–1^) than trimethoprim (1–15 ng L^–1^). Some review articles focusing on the occurrence of pharmaceuticals in African waters have reported ibuprofen, sulfamethoxazole, and efavirenz as some of the most prominent drugs in water.^21,22^

### 3.3 Comparisons of Detected Concentrations in Seawater with Other Studies

Table 3 shows the maximum concentrations detected for the investigated pharmaceuticals in seawater samples around the world in comparison with the results of the present study. Among other investigated pharmaceuticals, it was observed that efavirenz was the least monitored drug in seawater. This is expected in countries that are located outside the borders of the African continent where this drug is highly prescribed to HIV-positive people. As a result, the studies on environmental monitoring of efavirenz are more intense in South Africa and Kenya than anywhere in the world.^23^ However, this narrative should change considering that this antiretroviral drug has been detected in the coastal waters of Belgium.^24^ Even in the two African countries (South Africa and Kenya) where efavirenz is constantly detected in water systems, its monitoring in seawater has been neglected. In this context, the present study shows the presence of efavirenz in seawater samples from East London, thereby showcasing a need to include efavirenz on the list of pharmaceuticals that need to be monitored in marine and coastal environments.

**Table 3:** Concentrations of Investigated Pharmaceuticals Detected in Seawater across the Globe^a^

study site	detected maximum concentration in seawater (ng L^–1^)					reference
	NSAIDs		antibiotics		antiretroviral	
	ibuprofen	naproxen	sulfamethoxazole	trimethoprim	efavirenz	
Marmara Sea, Turkey	2130	340				(25)
Portuguese coast, Portugal				56		(26)
Portuguese coast, Portugal	222	178				(27)
Belgian Part of the North Sea		57			1	(24)
Red Sea, Saudi Arabia	509		62	66		(28)
Jiaozhou Bay, North China			0.0004			(29)
Kuwait’s coastal waters, Kuwait	4206	489	571	187		(30)
Antarctic		D		<0.1		(31)
Durban, South Africa	160	160				(17)
Cape Town, South Africa			4.79			(19)
**East London, South Africa**	90	57	30	15	76	**this study**

a D means the pharmaceutical was detected, but its amount was not reported.

The reviewed literature shows the investigated NSAIDs as more detected pharmaceuticals in seawater compared to antibiotics (Table 3). This has always been a general observation with more researchers interested in environmental monitoring of NSAIDs due to their easy accessibility as they can be accessed from medication dispensers without producing proof of sickness from any healthcare provider. In comparison with studies conducted in other countries (Table 3), the two NSAIDs under evaluation had lower concentrations in South Africa which is not in agreement with previous observations where it was established that the levels of pharmaceuticals in the African waters exceed those from the European countries.^22^ The same observation was made in most cases for antibiotics (Table 3). Based on the provided summary in Table 3, it is evident that the levels of the investigated pharmaceuticals found in the East London coastal environment are mostly on the lower side of concentration ranges detected globally. This could be due to massive dilutions of these pharmaceuticals occurring when they enter the oceans, which is the same situation that is experienced across the globe. Sampling can also play a role in the concentrations found in this study, as the collection of samples did not take into consideration any episodic events and the seasonal influence was not considered.

### 3.4 Health Risk Assessment and Future Considerations

As a measure of environmental risk assessment, the risk quotient values were computed as the ratio of the maximum concentration found for each pharmaceutical (Table 2) and the predicted no-effect concentration (PNEC) adopted from the literature.^15,21,32^ According to the literature, the high, medium, and low risk for a test organism are indicated by a risk quotient value equal to ≥1, 0.1 to 1, and <0.1, respectively.^21^ Based on the risk quotient values attained (Table 4) by considering the maximum detected concentrations (57 ng L^–1^) of naproxen and ibuprofen (461 ng L^–1^), low risks toward various species such as *Vibrio fischeri*, *Daphnia magna*, algae, and fish were observed. In the case of efavirenz, the applied PNEC value of 0.130 μg L^–1^ was taken from elsewhere^21^ and used as an indication of risk assessment toward algae, crustaceans, and fish. In this case, a resultant risk quotient value was 4.4 indicating high risk. Upon applying a similar approach for trimethoprim, a negligible risk was observed for algae and fish. For a different antibiotic, sulfamethoxazole, high risk was only observed in the case of algae where the risk quotient value was 22.2. This observation is different from the results presented in a different South African-based study where a risk quotient was 0.15 for sulfamethoxazole in stream water.^15^ These results show that only efavirenz and sulfamethoxazole resulted in a high risk. This further highlights a need to closely monitor the release of efavirenz and sulfamethoxazole into waterbodies as these pharmaceuticals have been the subject of numerous investigations in South Africa due to their constant presence in both wastewater and surface water.^2,33^ Trimethoprim might have been observed to present negligible risk in this study, but its constant presence in South African waters as indicated elsewhere^15,34^ is of concern.

**Table 4:** Health Risk Assessment Based on the Maximum Detected Concentrations

pharmaceutical	maximum detected concentration (μg L^–1^)	PNEC (μg L^–1^)			risk quotient				
		Vibrio fischeri	Daphnia magna	algae	fish	Vibrio fischeri	Daphnia magna	algae	fish
naproxen	0.057	21.2^a^	25^a^	626^a^	34^a^	0.003	0.002	0.000	0.002
ibuprofen	0.461	35.7^a^	9.06^a^	5.7^a^	170^a^	0.013	0.051	0.081	0.003
efavirenz	0.572	0.13^b^	4.4^b^						
trimethoprim	0.015			16^c^	100^c^			0.001	0.000
sulfamethoxazole	0.060		25.2^c^	0.0027^c^	562.5^c^		0.002	22.2	0.000

a PNEC values attained from ref (32).

b PNEC values from ref (21) and used as an indication of risk assessment toward algae, crustaceans, and fish.

c PNEC values assessed from ref (35).

All pharmaceuticals under examination have been previously found present in South African waters.^6^ However, the extent of their occurrence in South African waterbodies and their fate have always been overlooked as most studies on their investigation focused largely on wastewater and surface water from inland sources. This study revealed an important aspect: the same pharmaceuticals found in inland waters are carried through the rivers into the estuaries and eventually make their way into the ocean. This showcases a need to monitor the occurrence of the same pharmaceuticals in seafood from large South African cities. Environmental monitoring studies are needed to investigate if there is continuous detection of pharmaceuticals in the coastal environment. This is important to perform the risk assessment as the pharmaceuticals investigated in this study have been previously found to present risk toward aquatic organisms in previous South African-based investigations.^21,32^

### 3.5 Suspect Screening of Pharmaceuticals in Water Samples

The 57 compounds tentatively identified in the investigated water samples are listed in Table 5. Antibiotics were more dominant in the suspect list with this observation agreeing with South African-based studies which have shown the widespread of antibiotics in aquatic resources.^36^ The detection of NSAIDs and analgesics was also expected with these drugs being constantly detected in high amounts in South African waters.^32^ As shown in Table 6, the pharmaceuticals appearing prominently (detected in not less than 8 samples) in environmental samples were sulfadimethoxine, nafcillin (both antibiotics), atazanavir (antiretroviral drug), allopurinol (xanthine oxidase inhibitor), and acetaminophen (analgesic). While other pharmaceuticals are not commonly monitored in South African water bodies, acetaminophen is a common drug found in large quantities in South African environmental waters.^37,38^ Among the rivers, the Gonubie River (10 detected pharmaceuticals) was found to be the least contaminated while most of the pharmaceuticals (25 detected drugs) were detected in the Quenera River. In the Buffalo River, 20 pharmaceuticals were positively identified. Recreational activities could contribute to the number of pharmaceuticals found in Quenera and Gonubie rivers. On the other side, the Buffalo River is already known for its contamination with chemicals such as pharmaceuticals,^20^ pesticides,^12^ and other organics.^39,40^ The contamination of this river could be linked to various sources and activities as the Buffalo River flows through King William’s Town, Mdantsane Township, and other various communities. In addition, several WWTPs, with most of them not efficiently operational, discharge their effluent into the Buffalo River.^41^ Therefore, this study clearly indicates that pharmaceuticals are pumped into the Buffalo River and flow into its estuary, where they are released into the Indian Ocean. Table 6 further shows the distribution of pharmaceuticals in seawater. These findings suggest a need to perform a detailed analysis and quantitation of the reported pharmaceuticals in a coastal and marine environment.

**Table 5:** List of Pharmaceuticals Detected through the Suspect Screening Process in the Investigated Samples

class	pharmaceuticals	retention time (min)	score (%)	ionized mass (*m*/*z*)	fragment ion 1	fragment ion 2	fragment ion 3
antibiotics	1. Amoxicillin	4.71	97.2	366.111	349.085	211.071	114.001
	2. Azithromycin	11.19	89.2	750.532	533.080	256.054	124.083
	3. Cinoxacin	7.44	94.9	263.066	256.054	153.136	137.103
	4. Clindamycin	12.13	99.9	425.184	304.295	258.049	126.126
	5. Diaveridine	4.47	86.8	261.130	239.149	178.133	133.085
	6. Nafcillin	4.54	99.9	415.248	393.204	153.135	89.056
	7. Nitrofurantoin	4.51	99.9	239.040	207.120	123.089	95.082
	8. Novobiocin	8.34	85.2	613.246	406.188	219.107	125.062
	9. Metaflumizone	9.52	94.6	507.124	463.297	385.287	256.054
	10. Myriocin	8.87	90.8	402.285	357.255	313.229	151.091
	11. Ofloxacin	9.80	99.2	362.150	318.158	273.099	221.07
	12. Ormetoprim	11.17	85.6	275.147	259.116	229.106	123.073
	13. Rifampicin	11.84	99.9	823.404	791.787	519.217	399.162
	14. Rifaximin	8.56	99.9	786.462	764.458	605.351	546.651
	15. Sulfadiazine	6.43	98.1	251.059	209.057	153.135	137.103
	16. Sulfadimethoxine	10.31	99.9	311.078	282.275	256.056	137.104
	17. Sulfathiazole	11.61	87.3	256.015	239.233	137.104	124.083
	18. Tetracycline	8.85	89.3	445.159	428.134	427.149	410.123
antidepressant	19. Amitriptyline	11.76	99.9	278.042	256.059	137.107	104.106
antiretroviral drugs	20. Atazanavir	11.24	82.7	705.397	697.350	604.229	393.294
	21. Lopinavir	11.80	90.2	630.373	612.362	449.270	429.253
	22. Ritonavir	8.56	99.9	720.427	698.414	676.640	654.387
antidiabetic	23. Pioglitazone	9.21	98.7	357.261	313.234	269.208	104.107
	24. *N*-acetyl-sitagliptin (metabolite of sitagliptin)	5.19	98.3	450.275	428.262	406.249	362.224
antiallergic	25. Levocetirizine	5.89	99.5	389.247	315.174	256.056	153.135
	26. Terfenadine	5.20	97.8	472.288	450.275	439.253	406.249
cardiovascular agent	27. Allopurinol	1.44	80.0	137.046	128.046	110.035	81.045
	28. Atenolol	4.26	99.8	283.170	261.125	157.077	93.062
	29. Dipyridamole	5.22	99.9	505.292	483.279	472.288	428.262
	30. Dronedarone	12.36	99.8	557.312	441.292	393.292	256.054
	31. Digoxin	11.99	97.8	781.436	689.511	338.336	137.103
	32. Flecainide	4.54	80.7	416.148	399.121	371.223	344.190
	33. Irbesartan	9.67	85.2	429.239	385.286	256.054	104.102
	34. Lisinopril	5.16	99.9	406.249	395.227	384.236	318.196
	35. Norlidocaine (metabolite of lidocaine)	1.71	99.8	207.149	198.861	158.964	80.947
	36. Prazosin	5.15	99.9	384.236	362.223	340.210	318.197
	37. Oxymetazoline	4.47	99.9	261.130	239.149	178.133	89.089
	38. Vardenafil	8.80	86.4	489.228	445.309	357.257	313.231
NSAIDs and analgesic	39. Acetaminophen	1.33	81.6	152.070	110.060	92.050	65.0409
	40. Alfentanil	4.91	98.5	417.262	385.237	268.178	153.135
	41. Betamethasone	12.18	85.5	393.297	338.341	256.059	137.107
	42. Dexamethasone	4.48	84.0	393.205	373.200	355.18	237.126
	43. Diclofenac	5.36	97.2	296.021	250.018	215.049	112.028
	44. Fenoprofen	5.78	99.9	243.101	205.055	185.110	159.061
	45. Ketoprofen	10.87	98.2	255.055	239.232	207.119	124.083
	46. Mefenamic acid	7.32	94.9	296.214	271.183	145.117	127.107
	47. Piroxicam	7.64	99.9	332.325	313.177	256.054	104.104
	48. Sufentanil	1.66	98.7	387.210	272.944	151.035	112.006
	49. Sulfasalazine	10.35	98.5	399.073	311.250	289.268	71.082
steroids	50. Cinobufagin	9.90	97.8	443.328	341.260	297.234	107.067
	51. Estradiol	1.55	93.2	273.184	241.990	151.031	110.005
antiepileptics	52. Amisulpride	14.05	96.9	371.159	226.948	137.104	90.974
	53. Carbamazepine	5.89	97.6	237.117	215.135	152.013	88.072
	54. Clozapine	4.37	99.9	327.196	305.152	283.170	227.171
	55. Flurazepam	4.64	98.3	388.254	371.227	349.183	327.201
	56. Nitrazepam	10.72	99.9	282.279	256.059	166.097	137.107
	57. Lamotrigine	10.73	87.0	256.054	166.093	137.103	90.907

**Table 6:** Prevalence of Pharmaceuticals in Aqueous Samples

pharmaceutical (detection frequency)	rivers			estuaries		sea water					
	Buffalo	Quenera	Gonubie	Quenera	Gonubie	site 1	site 2	site 3	site 4	site 5	site 6
Acetaminophen (8/11)	√			√	√	√	√	√		√	√
Alfentanil (1/11)									√		
Allopurinol (10/11)	√	√		√	√	√	√	√	√	√	√
Atazanavir (9/11)	√	√	√	√	√	√		√	√	√	
Atenolol (2/11)						√				√	
Amitriptyline (1/11)		√									
Amisulpride (2/11)								√			√
Amoxicillin (4/11)		√					√			√	√
Azithromycin (3/11)		√		√		√					
Betamethasone (3/11)	√	√					√				
Carbamazepine (1/11)											√
Cinoxacin (1/11)									√		
Cinobufagin (1/11)						√					
Clindamycin (1/11)						√					
Clozapine (6/11)	√			√				√	√	√	√
Diaveridine (2/11)		√		√							
Diclofenac (2/11)								√			√
Dipyridamole (2/11)			√				√				
Digoxin (4/11)		√		√		√				√	
Dexamethasone (2/11)				√		√					
Dronedarone (5/11)			√	√		√		√		√	
Estradiol (6/11)	√			√	√	√	√	√			
Fenoprofen (1/11)				√							
Flecainide (1/11)									√		
Flurazepam (1/11)		√									
Irbesartan (8/11)	√		√	√	√	√	√	√		√	
Ketoprofen (6/11)		√	√		√				√	√	√
Mefenamic acid (7/11)	√	√			√	√	√	√			√
Metaflumizone (1/11)						√					
Myriocin (2/11)				√		√					
*N*-acetyl-sitagliptin (metabolite of sitagliptin) (3/11)	√					√			√		
Nafcillin (8/11)	√		√	√	√	√	√	√			√
Nitrofurantoin (6/11)	√		√				√	√	√	√	
Nitrazepam (2/11)		√		√							
Novobiocin (1/11)				√							
Norlidocaine (metabolite of lidocaine) (4/11)		√			√				√		√
Ofloxacin (5/11)		√		√		√			√	√	
Ormetoprim (2/11)		√		√							
Oxymetazoline (4/11)	√		√	√						√	
Lamotrigine (5/11)		√				√	√	√		√	
Lopinavir (4/11)	√		√				√	√			
Lisinopril (2/11)		√		√							
Levocetirizine (2/11)				√			√				
Pioglitazone (1/11)		√									
Piroxicam (1/11)						√					
Primidone (1/11)	√										
Rifampicin (5/11)				√	√	√			√		√
Rifaximin (1/11)		√									
Ritonavir (4/11)	√	√			√			√			
Sufentanil (1/11)		√									√
Sulfadiazine (3/11)	√						√				√
Sulfadimethoxine (9/11)	√	√		√	√	√	√	√	√	√	
Sulfathiazole (5/11)				√				√	√	√	√
Sulfasalazine (7/11)	√	√			√	√	√	√		√	
Terfenadine (3/11)		√		√			√				
Tetracycline (7/11)	√	√		√		√		√	√	√	
Vardenafil (7/11)	√		√	√		√	√	√	√		

## 4 Overall Remarks

Initially, this study investigated the occurrence of five pharmaceuticals belonging to three therapeutic classes in the coastal waters of East London, South Africa. Pharmaceuticals belonging to the classes of NSAIDs, antibiotics, and antiretrovirals are constantly detected in South African waterbodies. Therefore, this is an important investigation to determine the transfer of selected pharmaceuticals from inland waters to the coastal environment. All five pharmaceuticals under investigation were detected and quantified in East London coastal waters. It is of concern that there is a high health risk associated with the concentrations detected for efavirenz and sulfamethoxazole. The detection of these pharmaceuticals along the coast of East London means that the contamination in the study area is not far different from the observations of the previous studies conducted in the highly populated and larger cities of Durban and Cape Town, South Africa. This suggests that the smaller and less populated cities must also always be considered in the national surveys. This study further showcased a need to investigate the occurrence of a wide range of pharmaceuticals in East London coastal waters, as many additional drugs (57) were tentatively identified in the investigated samples.
